# Etiology of Phthisis

**Published:** 1881-04

**Authors:** 


DR. ED. LABBÉ ON THE ETIOLOGY OF PHTHISIS. (*L'Union Médicale*, February 1, 1881.—Abstract.)

I might cite numberless cases of acquired tuberculosis. I would simply need peruse a list of the various diseases ; thus, diabetes, cirrhosis, though more rarely, and other chronic diseases may be complicated with tuberculosis. All the various trades which oblige so many wretched beings to live in confined atmospheres, far from daylight, as in mines ; those which expose one to breathe metallic or other sorts of dusts ; furnish a large proportion of cases of that disease. Pregnancy, lactation, whooping cough, measles, prolonged dyspepsia, inanition from lesions of the œsophagus, can lead to tuberculosis. The rheumatic, cancerous, and syphilitic diatheses, are often complicated by tuberculosis, which induces their fatal termination. If all these are real causes, if we admit the identity of these various forms of tuberculosis, it may as well be granted that the constitutional disease scrofula, has a still more powerful predisposition to induce tuberculosis ; scrofulous diseases, at an early period, are already superimposed on an impaired constitution. No difference, clinical or histological, exists between pulmonary phthisis of a scrofulous origin and other forms of phthisis.

The tissue in which the tuberculosis has developed may impart to the morbid process a different course; in this manner, pulmonary phthisis progresses more rapidly than a tuberculosis localized in the testicle, or on the skin, yet the tubercular lesion is the same. It is useless to insist on the greater risk of dissemination from highly vascular organs, crowded with lymphatics; according to Laënnec, the softening of a tubercle is the most frequent cause of a general dissemination of the disease. We may add, that the rapidity of the softening and dissemination depends also on the exhaustion of the patient. To recapitulate: Let tuberculosis be local or general, it is always one and the same disease. The only specific character which could be ascribed to it would be its contagiousness, as is probable; but this would become the strongest argument against the diathetic character of tuberculosis.

As to hereditary descent, there is no doubt that a phthisical parent may start a phthisical progeny, but how few hereditary cases compare with those in which the disease is acquired! Not one in ten. Among the consumptive in my service, there is one in eight. We may refer the diseases to indirect inheritance (in this sense that a constitution congenitally weakened is more exposed to tuberculosis), but not the sort of inheritance observed in gout, in the *rheumic* diathesis (*dartre*), or in scrofulosis.

Scrofula, on the contrary, is most always inherited, and seldom acquired. Not a disease in itself, it is a predisposition to a group of maladies, differing by their seat and by their course, and identical as to their origin. In the same manner as one is a victim of the gouty diathesis before he has had gout, so one is scrofulous long before suffering from scrofulous swellings. But one is not tuberculous, because suffering from a dyspepsia, diabetes, or measles, though these might start pulmonary tuberculosis. Phthisis may terminate almost all the scrofulous affections, but it could not generate scrofula. Granting for a moment that the anatomo-pathological characteristics of scrofulous diseases consist in the tubercular granulation, the same difference remains between the diathesis (scrofula) and the disease (tuberculosis), as between the *rheumic* diathesis and eczema. Rheumic, scrofulous or artificial eczema, is always much alike; borrowing its difference from its etiology; the eczematous lesion is only one of the elements of the disease.

